# Chemical and Bioactivity Evaluation of* Eryngium planum* and* Cnicus benedictus* Polyphenolic-Rich Extracts

**DOI:** 10.1155/2019/3692605

**Published:** 2019-03-12

**Authors:** Gabriela Paun, Elena Neagu, Veronica Moroeanu, Camelia Albu, Simona Savin, Gabriel Lucian Radu

**Affiliations:** ^1^National Institute for Research-Development of Biological Sciences, Bucharest 6, 060031, Romania; ^2^Faculty of Applied Chemistry and Materials Science, Politehnica University of Bucharest, Bucharest, Romania

## Abstract

This study evaluated the biological activities of* Eryngium planum *and* of Cnicus benedictus* extracts enriched in polyphenols obtained by nanofiltration. The HPLC-MS analysis showed that* E. planum* contains mainly flavonoids, especially rutin, while in* C. benedictus* extracts show the high concentration of the phenolic acids, principally the chlorogenic acid and sinapic acid. Herein, there is the first report of ursolic acid, genistin, and isorhamnetin in* E. planum *and* C. benedictus. C. benedictus* polyphenolic-rich extract showed high scavenging activity (IC_50_=0.0081 mg/mL) comparable to that of standard compound (ascorbic acid) and a higher reducing power (IC_50_= 0.082 mg/mL), with IC_50_ having a significantly lower value than IC_50_ for ascorbic acid. Both extracts were nontoxic to NCTC cell line. Among the investigated herbs,* E. planum* polyphenolic-rich extract showed the highest inhibitory activities with the IC_50_ value of 31.3 *μ*g/mL for lipoxygenase and 24.6 *μ*g/mL for hyaluronidase. Both polyphenolic-rich extracts had a higher inhibitory effect on *α*-amylase and *α*-glucosidase than that of the acarbose. The synergistic effect of ursolic acid, rutin, chlorogenic acid, rosmarinic acid, genistin, and daidzein identified in polyphenolic-rich extracts could be mainly responsible for the pharmacological potentials of the studied extracts used in managing inflammation and diabetes.

## 1. Introduction

Recently, the research is more and more focused on the polyphenol's effect on the human health and on the natural antioxidants sources with low or no side effects to be used in medicine and in the food industry [1]. Data from literature firmly support the role of phenolic compounds in the prevention of degenerative diseases, such as neurodegenerative diseases and diabetes mellitus [2–4]. Several studies showed that the *α*-amylases and *α*-glucosidases have significant activities in the metabolism of polysaccharides. Dietary interventions based on plant natural inhibitors could successfully replace commercially drugs, such as acarbose, in the management of hyperglycaemia. The benefit stays in that there are no adverse side effects [5].

The frequent use of nonsteroidal anti-inflammatory drugs (NSAIDs) with various side effects determined lately a new direction in research toward the natural sources, effective against inflammatory disorders and with few side effects.

Lipoxygenases, which catalyse the addition of oxygen to arachidonic acid and other polyunsaturated fatty acids, and hyaluronidase, which hydrolyses hyaluronic acid, are involved in the inflammatory process [6, 7].

Development and use of efficient technologies for the bioactive compound's extraction and concentration represent a current priority. Nowadays, ultrasonic-assisted extraction (UAE) and membrane technology have become the most important and efficient technique for extraction [7, 8], separation, and concentration of the phenolic compounds from various plant sources [8–13].

*Eryngium planum L.* (Apiaceae family) is a medicinal plant used in European and Asian traditional medicine, especially for various inflammatory disorders treatment. The* Eryngium species* aerial part bioactivity is mainly generated by polyphenols and saponins [14–16].

*Cnicus benedictus* L. (blessed thistle) belonging to the family Asteraceae is mainly distributed from Asia Minor through southern Europe. This medicinal plant contains alkaloids, cnicin, benedictin, mucilage, polyacetylene, triterpenoide, lignans, flavonoids, tannin, phytosterines, and volatile oils [17–19]. This is a plant with antidepressive, anti-inflammatory, antiseptic, cardiac, and antimicrobial properties and cnicin is the compound responsible for blessed thistle's medicinal properties [20].

There are only several studies on the chemical composition of these herbs, yet very few phytopharmacological studies have been exhaustively investigated up till now. Taking into account the considerations mentioned above, the present study was undertaken to investigate the anti-inflammatory and the antidiabetic activities of* Eryngium planum* and* Cnicus benedictus* polyphenolic-rich extracts. In addition, the antioxidant activity and cytotoxicity of polyphenolic-rich extracts of selected herbs were also investigated.

However, up to date, these characteristics of* E. planum and C. benedictus* polyphenolic-rich extracts have not yet been studied; therefore, no reference is available regarding the UAE and the membrane concentration of polyphenolic compounds from these herbs.

## 2. Materials and Methods

### 2.1. Plant Material

*Eryngium planum* herb was collected from Valcea district (Romania) and* Cnicus benedictus *herb was collected from Razhdak district (Bulgaria). The voucher specimen number 657490 for* Eryngium planum* and voucher specimen number 665998 for* Cnicus benedictus* were preserved in the Botanic Garden of Cluj-Napoca, Romania.

### 2.2. Extraction and Processing

Dried aerial parts were grounded (10 g), mixed with the 50% (v/v) ethanol in water as solvent, and shaken for 5 minutes for the extraction process. Next, the extraction was performed by ultrasonic-assisted extraction (UAE) using a sonication water bath (Elma Transsonic T 460, Germany) at 35 kHz for 90 min. After these steps, extracts were filtered through the filter paper under vacuum and microfiltered through 0.45 *μ*m pore size microfiltration membrane (Millipore). Nanofiltration was applied to concentrate biologically valuable components and obtain polyphenolic-rich extracts. The nanofiltration was performed with a tangential filtration KOCH Membrane laboratory unit equipped with an NF90 (Dow Filmtec, Sterlitech Company, USA), with a molecular weight cut-off of 200–300 Da, made of polyamide material.

### 2.3. Total Phenolic and Flavonoid Content

The total phenolic content was determined using Folin-Ciocalteu assay [21]. The total phenolic content was calculated on the basis of a calibration curve of gallic acid (y=0.0027x+0.017, R^2^ = 0.9965) and expressed as mg gallic acid equivalents per litter of extracts (mg GAE/L).

The total flavonoid content was measured using the AlCl_3_ colorimetric method as described by Lin [22]. Results were expressed as quercetin (QE) mg/mL using the equation based on the calibration curve: y = 0.004x + 0.0535; R^2^ = 0.9934.

### 2.4. HPLC-MS Analysis

The analysis of polyphenols was performed using a validated HPLC-MS method [23]. All solvents and reference compounds (p-coumaric acid, caffeic acid, ferulic acid, gallic acid,* chlorogenic* acid, rosmarinic acid, ellagic acid, rutin, luteolin, quercetin, quercetin 3-*β*-D-glucoside, apigenin, kaempferol, daidzein, genistein, and genistin) were purchased from Sigma (Sigma Aldrich, Germany), Fluka (Switzerland), and Roth (Carl Roth GmbH, Germany).

The liquid chromatography analysis was performed using a HPLC SHIMADZU system coupled to a MS detector, LCMS-2010 detector (liquid chromatography mass spectrometer), and equipped with an electrospray ionization (ESI) interface. For analysis, a C18 Kromasil 3.5, 2.1x100mm column was used. The mobile phase was composed of solvent A (formic acid in water, pH=3.0) and solvent B (formic acid in acetonitrile, pH=3.0). The polyphenolic compound's separation was performed using binary gradient elution: 0 min 5% solvent B; 0.01-20 min 5-30% solvent B; 20-40 min 30% solvent B; 40.01-50 min 30-50% solvent B; and 50.01-52 min 50-5% solvent B. The flow rate was 0-5 min 0.1 mL/min; 5.01-15 min 0.2 mL/min; 15.01-35 min 0.1 mL/min; 35.01-50 min 0.2 mL/min; and 50-52 min 0.1 mL/min. The quantitative analysis of the components was achieved with reference to standards.

For ursolic acid HPLC-MS determination, a C18 Nucleosil 100-3.5, 100x2.1 mm, KROMASIL column was used. Mobile phase was formic acid in water (solvent A) and methanol (solvent B). The flow rate for solvent A was 0.042 mL/min and for solvent B was 0.258 mL/min. The injection volume was 20 *μ*L and the temperature of the column was kept constant at 20°C. ESI source and negative ionization mode were used.

### 2.5. Antioxidant Activity

#### 2.5.1. DPPH Radical Scavenging Activity

The 2,2-diphenyl-1-picrylhydrazyl (DPPH) radical scavenging activity of the polyphenolic-rich extracts was performed according to the method described by Bondet with some modification [24]. 100* μ*l sample extract or standard (ascorbic acid) with varying concentrations was mixed with 2900 mL of DPPH*∙* methanolic solution and the absorbance was measured at 519 nm after 30 min of reaction.

#### 2.5.2. Reducing Power

The reduction of Fe^3+^ into the Fe^2+^ potential of the extracts was determined according to the method of Baba and Malik [25]. In brief, 100 *μ*L of extract, 2.5 mL phosphate buffer (0.2M, pH 6.6), and 2.5 mL potassium ferricyanide (1%) were mixed and incubated at 50°C for 20 min. Then, 2.5 mL trichloroacetic acid (10%) was added, the mixture was centrifuged, and the upper layer (2.5 mL) was mixed with 2.5 mL of deionized water and 0.5 mL of 0.1% ferric chloride; then the absorbance was measured at 700 nm; ascorbic acid was used as standard.

### 2.6. *In Vitro* Anti-Inflammatory Assay

#### 2.6.1. Lipoxygenase (LOX) Inhibition

The LOX inhibition was performed by the spectrophotometric assay [26]. The increase in absorbance at 234 nm was due to the formation of the product 13-hydroperoxyocta-decadienoic acid from the reaction of oxygen and linoleic acid catalysed by lipoxygenase. For the blank solution, 100 *μ*L LOX solution 2200 U/mL was mixed with 100 *μ*L linoleic acid and 3050 *μ*L boric acid buffer 0.2 M (pH 9.0). For samples, 50 *μ*L of borate buffer was replaced with 50 *μ*L of the extract. The mixture was incubated for 15 min; then 100 *μ*L linoleic acid was added to the mixture and the absorbance was read at 234 nm for 2 minutes. The % inhibition for different concentrations of the extracts was calculated as

(1) $$\begin{array}{c} I\left(\;\%\;\right)=\left(\;1-\frac{\Delta A_{s}/\Delta t}{\Delta A_{ctr}/\Delta t}\;\right)\times 100 \end{array}$$

where A_s_ and A_ctr_ are the increase of absorbance for the sample extracts and the buffer instead of inhibitor (sample). Ibuprofen is used as standard.

#### 2.6.2. Hyaluronidase (HYA) Inhibition

The hyaluronidase inhibition was determined by the method described by Sahasrabudhe and Dedhar with slight modifications [27]. Briefly, 100 *μ*l of hyaluronidase (type-1-S from bovine, 400 units/mL) in acetate buffer (0.1 M, pH 3.5) and 50 *μ*L of plant extract was incubated for 20 min at 37°C; then 100 *μ*L of CaCl_2_ solution 12.5 mM was added and the mixture was incubated also at 37°C for 20 minutes. Then, the reaction was initiated by adding 250 *μ*L of sodium hyaluronate (1.2 mg/mL) and incubated at 37°C for 40 min; 100 *μ*L of sodium hydroxide solution 0.4 M and 100 *μ*L of potassium borate (0.4 M) were added to the reaction mixture and incubated for 3 min in a boiling water bath. After cooling, 3000 *μ*L of 10% *p*-dimethylaminobenzaldehyde reagent was added and after 20 minutes the increase in absorbance was measured at 585 nm. The percentage of enzyme inhibition was calculated as

(2) $$\begin{array}{c} I\left(\;\%\;\right)=\left(\;1-\frac{\Delta A_{s}}{\Delta A_{ctr}}\;\right)\times 100 \end{array}$$

where A_s_ and A_ctr_ are the absorbance of the sample extracts and absorbance of control (buffer instead of inhibitor). Ibuprofen is used as standard.

### 2.7. In Vitro Antidiabetic Assays

#### 2.7.1. Inhibition of *α*-Amylase Activity

The *α*-amylase inhibitory activity was evaluated according to Ranilla et al. with slight modification [28], as a previous reported paper [29]. In brief, the plant extract and *α*-amylase from hog pancreas solution (0.5 mg/mL) were incubated at 37°C for 20 min. After that, a 1% starch solution was added and incubated at the same temperature for 30 min. The reaction was stopped with dinitrosalicylic acid colour reagent. The mixture was incubated in a boiling water bath for 5 min, cooled to room temperature, and diluted with distilled water and the absorbance was recorded at 540 nm. Acarbose was used as positive control. The rate of enzyme inhibition was calculated according to the following equation:

(3) $$\begin{array}{c} I\left(\;\%\;\right)=\left(\;1-\frac{\Delta A_{s}}{\Delta A_{ctr}}\;\right)\times 100 \end{array}$$

where *A*_ctr_ is the absorbance of the control (without extract/acarbose) and *A*_s_ is the absorbance of the sample extract.

#### 2.7.2. *α*-Glucosidase Inhibition Assay

*α*-Glucosidase inhibition was evaluated using the procedures described in the literature [30]. Sample solution (50 *μ*L) was preincubated with 125 *μ*L of *α*-glucosidase from* Saccharomyces cerevisiae* (0.5 U/mL in phosphate buffer solution, pH 6.9) and 700 *μ*L of phosphate buffer at 37°C for 15 minutes. Then, 125 *μ*L of 5 mM p-nitrophenyl glucopyranoside (pNPG) was added to quench the reaction and the mixture was kept at 37°C for 30 minutes. The reaction was stopped by adding 1000 *μ*L of 0.2M Na_2_CO_3_ and the absorbance was recorded at 405 nm. Control sample was prepared to contain buffer instead of enzyme. Acarbose was used as positive control.

(4) $$\begin{array}{c} Inhibition\left(\;\%\;\right)=\left(\;\frac{A_{blank}-A_{sample}}{A_{blank}-A_{control}}\;\right)\times 100 \end{array}$$

where A_blank_ stands for the absorbance of the mixture without extract samples; A_sample_ stands for the absorbance of polyphenolic-rich extract samples; A_control_ stands for the absorbance of the mixture without a-glucosidase.

IC_50_ value (concentration of phenolic-rich extracts required to inhibit 50% of the enzyme activity) was calculated graphically by dose-dependent inhibition.

### 2.8. Cytotoxicity

The cell line of mouse fibroblast (NCTC clone 929 from the European Collection of cell Culture, Sigma-Aldrich, USA) was used to determine the cell viability by the MTT test. Fibroblast cultures (NCTC) were grown in MEM containing 10% fetal bovine serum (FBS) and PSN. The NCTC cell line was inoculated at 4 x 10^4^ cells/mL density. The cells cultured in standard conditions adhered to plastic after 24 hours of incubation; then the medium of culture changed with medium containing various concentrations of plant extracts (100, 250, and 500 *μ*g/mL). The plates with 96 wells were incubated for 24 and 72 hours at 37C in 5% CO_2_ air atmosphere. The culture control was untreated cells cultivated in MEM and 10% FBS, and the positive control was H_2_O_2_ (2*μ*L/mL). After incubation with MTT solution (3 hours, at 37°C), the plates were put on a shaker for 15 min and the absorbance was read at 570 nm on a microplate Mithras LB 940 (Berthold Technologies).

For the morphology, the culture of mouse fibroblast cells (NCTC), fixed in methanol and Giemsa stained, was observed after 72 hours after the addition of the extracts and acquired with Zeiss AxioStar Plus microscope (Carl Zeiss, Germany).

### 2.9. Statistical Analysis

All values are expressed as the mean ± standard deviation and analyzed using Microsoft Excel. Values were considered significant when p value is lower than 0.05.

## 3. Results and Discussion

### 3.1. Phytochemical Composition and Antioxidant Activity

Polyphenols (phenolic acids and flavonoids) are compounds exhibiting strong antioxidant activity and are responsible for these plants' medicinal properties. The nanofiltration process (NF) was used for the polyphenol's concentration to obtain the polyphenolic-rich extracts in the present study. The extracted fractions were evaluated by means of total phenolic compound (TPC), total flavonoid content (TFC), and antioxidant capacities (DPPH and reducing power assays) (Table 1).

The results indicated a good concentration performance of the membrane processes, resulting in a significant increase of the polyphenols contents in the nanofiltrate fractions.* C. benedictus* nanofiltrate retentate was found to have the highest TPC level (2.659 ± 0.128 mg GAE/mL), of which over 17.5% (0.478 ± 0.045 mg QE/mL) can be attributed to the total flavonoids.

**Figure 1:**
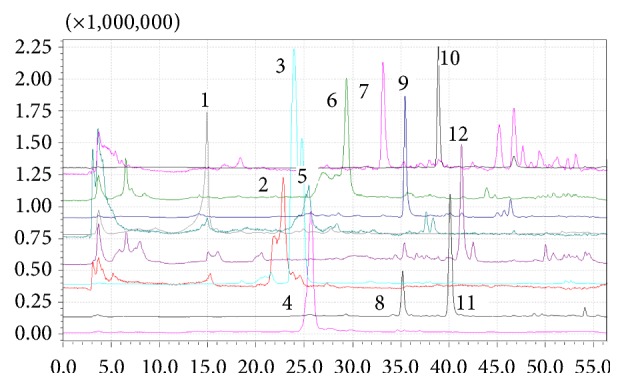
HPLC-MS chromatograms of the* Eryngium planum* polyphenolic-rich extract (1, chlorogenic acid, 2, p-coumaric acid, 3, rutin, 4, quercetin 3-*β*-D-glucoside, 5, ferulic acid, 6, rosmarinic acid, 7, daidzein, 8, luteolin, 9, quercetol, 10, genistein, 11, kaempferol, and 12, isorhamnetin).

**Figure 2:**
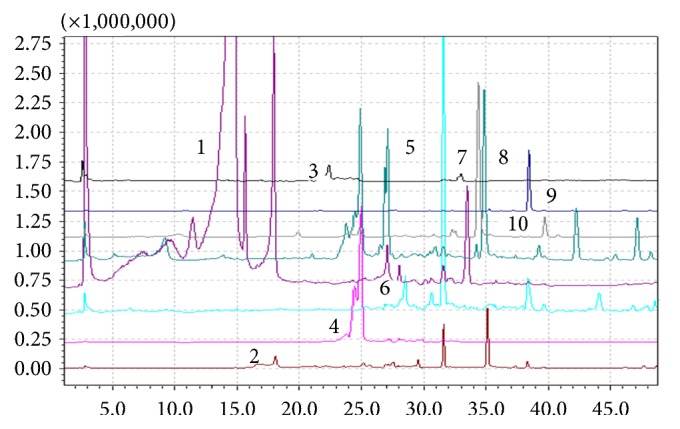
HPLC-MS chromatograms of the* Cnicus benedictus* polyphenolic-rich extract (1, chlorogenic acid, 2, caffeic acid, 3, p-coumaric acid, 4, sinapic acid, 5, ellagic acid, 6, rosmarinic acid, 7, luteolin, 8, quercetol, 9, genistein, and 10, kaempferol).

The polyphenolic compounds present in the MF and NF retentate fractions of* E. planum* and* C. benedictus* extracts were determined by HPLC-MS and the data presented in Table 2 can be correlated with Figures 1 and 2.

The HPLC-MS results showed that the extracts from* E. planum* contain mainly flavonoids, especially rutin and isoquercitrin, while in* C. benedictus* extracts show the high concentration of the phenolic acids, especially the chlorogenic acid and sinapic acid, in agreement with data reported in the literature [14, 31].

Our study's significant contribution consists of the identification and quantifying of new polyphenols for the studied herbal extracts. However, this is the first time that genistin and isorhamnetin are reported in* E. planum* and* C. benedictus *extracts.

In the studied extracts, ursolic acid, a triterpene responsible for many pharmacological effects, was identified and quantified also. Ursolic acid was concentrated from 76.77 *μ*g/mL to 158.04 *μ*g/mL in* E. planum* extracts and from 46.84 *μ*g/mL to 146.50 *μ*g/mL in* C. benedictus* extracts.

Antioxidant activity has been studied also, due to the fact that oxidative stress is considered to be the key factor in the anti-inflammatory process and in the pathogenesis of diabetic complications. Screening the antioxidant activity by DPPH and reducing power assays showed that for both plants extracts the nanofiltration retentates are more effective.

Table 1 shows that* C. benedictus* nanofiltrate retentate had the high scavenging activity (IC_50_ = 0.0081 mg/mL) comparable to that of ascorbic acid (IC_50_ = 0.0012 mg/mL) and a higher reducing power (IC_50_ = 0.082 mg/mL), with IC_50_ value significantly lower than that of the ascorbic acid (0.125 mg/mL), used as control.* E. planum* nanofiltrate retentate had a similar reducing power to control but a lower scavenging activity comparable to that of ascorbic acid. A significant correlation (p< 0.05) was found between antioxidant activity and polyphenol and flavone contents (Table 1S from supplementary material), indicating a significant contribution of these compounds to the antioxidant activity for these polyphenolic-rich extracts.

The bioactive compounds from herbal extracts concentrated by ultrafiltration (UF) and nanofiltration (NF) have been recently investigated as an alternative solution to the conventional methodologies. Paun and collaborators tested the ultrafiltration membrane for the concentration of antioxidants from* Phyllitis scolopendrium *L. extract [32]; Tylkowsky et al. investigated the NF process using with membranes with MWCO in the range of 200–500 Da for the treatment of ethanolic extracts from* Sideritis *spp. L. in order to concentrate polyphenols [33]; Conidi and collaborators concentrated clarified artichoke wastewater using a NF membrane (NF 270, polyamide) with MWCO of 200–300 Da [34]. All these researches proved the efficiency of both ultrafiltration and nanofiltration procedures to recover and concentrate polyphenols from different herbal extracts.

### 3.2. *In Vitro* Anti-Inflammatory Activity

Several enzymes have been stated as mediators of inflammation and appear to be involved in inflammatory disorders. Among them, lipoxygenases and hyaluronidases were used as a criterion of anti-inflammatory potential [35].* In vitro* anti-inflammatory activity of the* E. planum *and* C. benedictus* polyphenolic-rich extracts against lipoxygenase (LOX) and hyaluronidase (HYA) (Table 3) has been tested.

All studied extracts had inhibitory effects on the tested enzymes. Among the fractions, the* E. planum* polyphenolic-rich extract exhibited the highest inhibitory activity with IC_50_ value of 31.3±2.1 *μ*g/ml for LOX and 24.6±1.5 *μ*g/ml for HYA.

The phenolic acids, flavonoids, and isoflavones from plants were reported to have anti-inflammatory activity [36–39]. Therefore, the anti-inflammatory properties of* E. planum* and* C. benedictus* polyphenolic-rich extracts may be assigned to the synergic effect of ursolic acid and polyphenols such as rutin, chlorogenic acid, rosmarinic acid, genistin, and daidzein previously investigated for anti-inflammatory effects [36–39].

Ursolic acid present in high quantities in the concentrated extracts is of particular interest due to antioxidant, antimicrobial, anti-inflammatory, and hypoglycemic activities [40].

### 3.3. *α*-Amylase and *α*-Glucosidase Inhibition

Due to their inhibition capacity against *α*-amylase and *α*-glucosidase, these medicinal plant extracts provide a therapeutic approach, aiming at postprandial hyperglycemia reducing and controlling on diabetes disease [41]. Acarbose is an inhibitor of carbohydrates digesting enzymes used to decrease the glucose absorbance, but it has some gastrointestinal side effects [42]. A wide range of medicinal plants could be related to the inhibition of carbohydrate enzymes; therefore lately the studies on new *α*-amylase and *α*-glucosidase natural inhibitors are extended. [43].

The present study investigating enzyme's inhibitory potential of the selected herbs is the first report on the *α*-amylase and *α*-glucosidase inhibitory effects of* E. planum *and* C. benedictus* polyphenolic-rich extracts. The results about the interaction of the* E. planum *and* C. benedictus* polyphenolic-rich extracts with *α*-amylase and *α*-glucosidase are presented in Table 4.

All of the tested extracts inhibited *α*-amylase and *α*-glucosidase in a dose-dependent manner. As it can be observed in Table 4, both of the polyphenolic-rich extracts had higher inhibitory effect on *α*-amylase (IC_50_ = 8.27 *μ*g/mL, resp., IC_50_ = 1.2 *μ*g/mL) than the acarbose (IC_50_ = 17.68 *μ*g/mL).

In this study on the *α*-glucosidase inhibition, our data revealed that all the herbal extracts have stronger activity than acarbose (used as a standard inhibitor). The best *α*-glucosidase inhibition was determined in* E. planum* polyphenolic-rich extract (NF retentate) with IC_50_ of 5.94 *μ*g/mL, where the *α*-glucosidase content was almost 46 times lower than its value in case of using acarbose (272.58* μ*g/mL).

The data revealed that both polyphenolic-rich extracts (NF retentate) had the highest inhibitory activity, correlating with their total phenol and flavonoid high contents.

The presented results may provide fundamental data for further studies in designing new plant-based drugs or nutraceuticals. The phenolic compounds (phenolic acids and flavonoids) found in these extracts exert their protective properties against diabetes and these compounds were mentioned previously as inhibitors of *α*-amylase and *α*-glucosidase [44]. According to the previous findings, ursolic acid, a triterpene, has a significant inhibitory activity to *α*-amylase and *α*-glucosidase and therefore might play an important role in the treatment of diabetes mellitus and its complications [45]. The high contents of ursolic acid, rutin, and chlorogenic acid from* E. planum *and* C. benedictus* polyphenolic-rich extracts might be considered responsible for their *α*-amylase and *α*-glucosidase inhibition capacity.

For efficient therapeutic use in the treatment of hyperglycaemia, these natural inhibitors are preferred to have mild inhibitory activity against *α*-amylase and strong *α*-glucosidase inhibitory activity, because inhibition of both enzymes could have side gastrointestinal effect [46]. In support of this idea,* E. planum* polyphenolic-rich extract can be considered more relevant in the management of diabetes mellitus.

On the other hand, previously studies established that chronic inflammation is linked with type II diabetes and therefore targeting inflammation may ameliorate diabetes. Results presented in the paper showed that* E. planum* polyphenolic-rich extract has the highest anti-inflammatory activity, correlating with the highest antidiabetic activity based on the enzyme's strong inhibition.

### 3.4. Cytotoxicity

For the further therapeutic application of the medicinal plant extracts in discussion, the effect of* E. planum *and* C. benedictus* polyphenolic-rich extracts on the NCTC clone 929 stabilized cell line was tested. The toxicity of each extract was determined to carry out both quantitative and qualitative methods.

**Figure 3:**
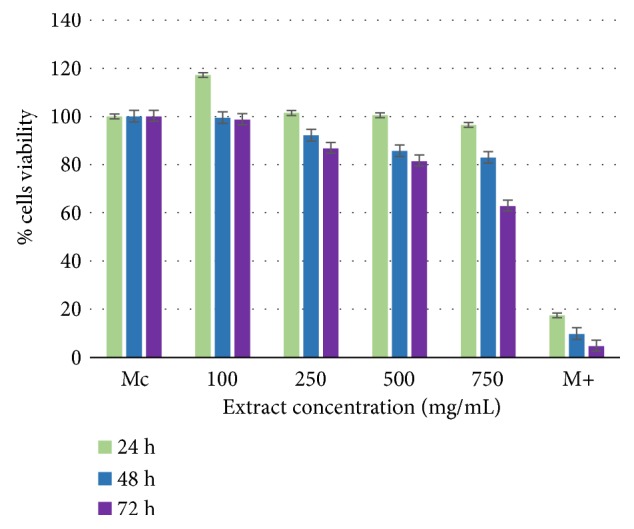
Cell viability of NCTC clone 929-line cells with* E. planum* polyphenolic-rich extract.

**Figure 4:**
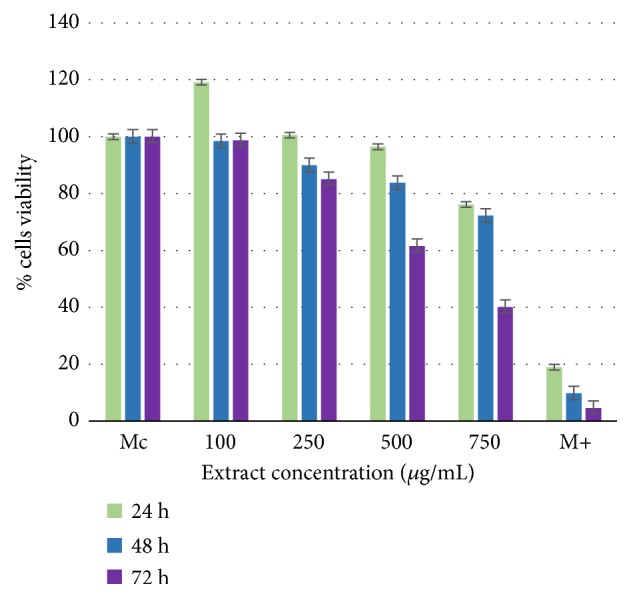
Cell viability of NCTC clone 929-line cells with* C. benedictus* polyphenolic-rich extract.

The* E. planum *polyphenolic-rich extract tested on NCTC line produced proliferative effect after 24 h, noncytotoxic effect after 48 h and 72 h within the concentration range of 100-500 *μ*g/mL, and a slightly cytotoxic effect at 750*μ*g/mL after 72 h (Figure 3). Cytotoxicity analysis for the* C. benedictus* polyphenolic-rich extract showed a noncytotoxic effect after 24 h and 48 h, but after 72 h this extract was slightly cytotoxic starting at 500 *μ*g/mL and moderately cytotoxic at 750 *μ*g/mL (Figure 4).

These data determined the optimal range of noncytotoxic concentrations for each of the plant extracts: up to 500 *μ*g/mL for* E. planum* and 250 *μ*g/mL for* C. benedictus*.

There are no references for the cytotoxic activity on fibroblasts for* Eryngium planum *and for* Cnicus benedictus*, but other* Eryngium* and* Cnicus* species were tested on various cancer cell lines [47–49].

**Figure 5:**
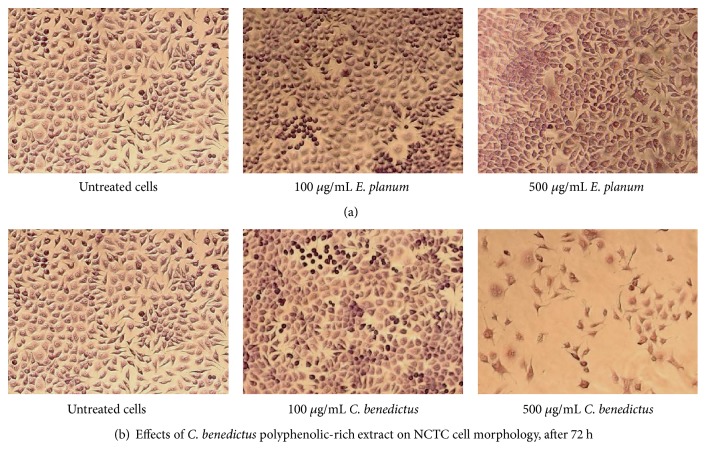


The cytotoxic effect of the analyzed extracts against the cell lines is presented in the photomicrographs (Figure 5). In both of the cases implying untreated cells (Figure 5(a), left) and the cells treated with 100 *μ*g/mL and 500 mg/L concentrations of* E. planum* polyphenolic-rich extract, no morphology or cell density changes could be noticed (Figure 5(a)). Cells were homogeneously distributed on the plate and exhibited the typical spindle-shape morphology of normal fibroblasts.

Withal, untreated cells (Figure 5(b), left) and cells treated with 100 *μ*g/mL of* C. benedictus* polyphenolic-rich extract did not exhibit morphological and cell density changes (Figure 5(b), middle), whereas for the cells treated with the same extract at 500 mg/L concentrations an alteration in cell morphology and decreased cell density were noticed, indicating a moderate cytotoxic effect (Figure 5(b), right).

## 4. Conclusions

In conclusion, this is the first report about the determination of ursolic acid, genistin, and isorhamnetin in* E. planum *and* C. benedictus* and also the first study to evaluate the* E. planum and C. benedictus* polyphenolic-rich extracts potentially used in therapy for hyperglycaemia.

The study results demonstrated that* Eryngium planum *and* Cnicus benedictus *extracts obtained by ultrasound-assisted extraction and concentrated by nanofiltration lead to obtaining an enriched fraction of bioactive polyphenolic compounds, consequently preserving a high antioxidant activity.

Both polyphenolic-rich extracts exhibited inhibition of the enzymatic activity relevant to type II diabetes management and anti-inflammatory correlation with a high content of ursolic acid and polyphenolic compounds. Moreover, phenolic acids and flavonoids were found to be mainly responsible for these bioactivities of the extracts according to Pearson's correlation coefficients. These findings provide new support for the traditional anti-inflammatory and antidiabetic application of* E. planum *and* C. benedictus *and indicate that both* polyphenolic-rich extracts could be utilized* as potential antidiabetic and anti-inflammatory agents.

## Figures and Tables

**Table 1:** The active biological compounds content and antioxidant activity of extract fractions.

Medicinal herb	Fraction	TPC,	TFC,	DPPH,	Reducing power,
		mgGAE/mL	mgQE/mL	EC_50_, mg/mL	EC_50_, mg/L
*E. planum*	MF	0.749±0.062	0.176±0.014	0.316±0.034	0.209±0.020
	NF retentate	1.882±0.104	0.378±0.035	0.197±0.031	0.128±0.012
*C. benedictus*	MF	1.847±0.134	0.223±0.020	0.711±0.042	0.140±0.012
	NF retentate	2.659±0.128	0.478±0.045	0.0081±0.0005	0.082±0.006
Ascorbic acid				0.0012±0.0001	0.125±0.010

MF, microfiltration process; NF, nanofiltration process.

Antioxidant activity is expressed as half inhibitory concentration (EC_50_); values are expressed as mean ± standard deviation of three measurements.

**Table 2:** HPLC-MS polyphenolic profile of extracts fractions.

Class	Compound [M/z]^−^	*Eryngium planum*		*Cnicus benedictus*	
		*µ*g/mL		*µ*g/mL	
		MF.	Conc.	MF.	Conc.
Flavonols	Rutin [609]	183.63	290.52	1.87	3.56
	Kaempferol [285]	3.55	4.20	0.62	0.73
	Isoquercetin [463]	23.40	36.11	7.54	9.75
	Isorhamnetin [477]	0.17	0.47	-	-
	Quercetol [301]	2.06	3.03	-	-
Flavone	Luteolin [285]	0.72	0.93	0.50	0.62
	Apigenin [269]	-	-	0.20	0.56
Phenolic acids	Caffeic acid [19]	4.51	5.68	4.07	5.58
	Rosmarinic acid [359]	15.53	18.95	7.62	10.17
	Ferulic acid [193]	6.39	7.07	-	-
	Chlorogenic acid [353]	40.33	63.02	315.11	454.29
	p-Coumaric acid [163]	4.29	4.66	7.52	8.35
	Sinapic acid [223]	0.67	9.93	109.74	249.85
Ellagitannins	Ellagic acid [301]	1.29	1.32	1.19	1.54
Isoflavones	Genistein [269]	1.42	1.49	2.20	2.47
	Genistin [433]	2.09	2.80	1.80	2.61
	Daidzein [253]	0.17	0.35	2.31	4.68

“-”, under limit of detection.

**Table 3:** Lipoxygenase and hyaluronidase inhibitory activity of *E. planum and C. benedictus* extract fractions.

Extract fractions		IC_50_, *µ*g/ml	
		LOX	HYA
*E. planum *extract	MF	104.8±9.6	37.9±2.1
	NF retentate	31.3±2.1	24.6±1.5
*C. benedictus *extract	MF	115.1±7.2	125.9±8.4
	NF retentate	52.7±3.4	93.6±7.0
Ibuprofen		69.7±0.005	14.3±0.8

Data are expressed as mean ± SD of triplicate determinations.

**Table 4:** IC_50_ values (*μ*g/ml) of *α*-amylase and *α*-glucosidase inhibition activity of polyphenolic-rich extracts.

Extract fractions		*α*-amylase	*α*-glucosidase
*E. planum *extract	MF	10.72 ± 0.8	24.99 ± 1.8
	NF retentate	8.27 ± 0.5	5.94 ± 0.35
*C. benedictus *extract	MF	5.41 ± 0.26	84.83 ± 3.2
	NF retentate	1.2 ± 0.01	48.54 ± 2.6
Acarbose		17.68 ± 1.2	272.58 ± 5.4

## Data Availability

The data used to support the findings of this study are available from the corresponding author upon request.

## Supplementary Materials

Supplementary Materials The correlation between bioactive compounds, antioxidant activity, and enzymes inhibitory effects of extracts is presented in Table 1S.
