# Supplementary material for: Chemical and Bioactivity Evaluation of Eryngium planum and Cnicus benedictus Polyphenolic-Rich Extracts
Source: Biomed Res Int. 2019 Mar 12;2019:3692605. doi: 10.1155/2019/3692605 (PMC6434295; doi:10.1155/2019/3692605)
Supplement: Supplementary Materials — The correlation between bioactive compounds, antioxidant activity, and enzymes inhibitory effects of extracts is presented in Table 1S. [file 3692605.f1.docx]

**Table 1S.**  Pearson's correlation coefficients between assays

| Bioactive compounds | Antioxidant activity | | Enzyme inhibitory activity | | | | |  |
| --- | --- | --- | --- | --- | --- | --- | --- | --- |
|  | DPPH | RP | | LOX | HYA | α-Amyl | α-Gluc | |
| TPC | − 0.960^***^ | -0.892^***^ | | 0.933^***^ | 0.947^**^ | -0.956 | 0.957^*^ | |
| TFC | − 0.823^**^ | -0.829^***^ | | 0.802^***^ | 0.813^**^ | -0.794^*^ | 0.799^*^ | |

TPC: total phenolic content; TFC: total flavonoid content; DPPH: 2-diphenyl-1-picrylhydrazyl; RP: reducing power; LOX: lipoxygenase; HYA: hyaluronidase; α-Amyl: α-amylase; α-Gluc: α-glucosidase.

* Significant (p < 0.05).

** Very significant (p < 0.01).

*** Highly significant (p < 0.001).
